# African swine fever whole-genome sequencing—Quantity wanted but quality needed

**DOI:** 10.1371/journal.ppat.1008779

**Published:** 2020-08-27

**Authors:** Jan H. Forth, Leonie F. Forth, Sandra Blome, Dirk Höper, Martin Beer

**Affiliations:** Institute of Diagnostic Virology, Friedrich-Loeffler-Institut, Greifswald—Insel Riems, Germany; University of Colorado Denver, UNITED STATES

## Abstract

The pandemic spread of African swine fever virus (ASFV) genotype II (GTII) has led to a global crisis. Since the circulating strains are almost identical, time and money have been mis-invested in whole-genome sequencing the last years. New methods, harmonised protocols for sample selection, sequencing, and bioinformatics are therefore urgently needed.

## African swine fever—From a neglected exotic disease to a pandemic threat

For decades, African swine fever (ASF) was regarded as an exotic foreign disease of wild and domestic suids without global impact. However, in 1957, the world was proven wrong [1]. While globalisation progressed and intercontinental travel thrived, animals and their products were shipped around the world and, with them, stowaways like the causative viral pathogen of ASF, African swine fever virus (ASFV). During the first pandemic following 1957, ASF spread intercontinentally to numerous countries, including Portugal, Spain, France, Belgium, the Netherlands, Brazil, the Dominican Republic, Haiti, and Cuba, killing millions of pigs [1]. However, eradication was successful from all those regions with the exception of Sardinia, where ASF remains endemic until today [2].

In 2007, ASF re-emerged in Georgia and subsequently spread through the Caucasus and Eastern Europe, reaching the European Union (EU) in 2014 and Asia in 2018. Now, ASF is definitively on the winning track threatening Western Europe and wreaking havoc in Asia’s pig industry [3]. Shockwaves triggered by this ongoing event have been sent around the globe, and today, even industries that are only distantly related to pigs—e.g., production of anticoagulants, enzymes, and sweets—are affected [4,5]. Therefore, the need for knowledge about this exotic pathogen as well as prediction and intervention strategies have become very urgent. One part of this knowledge is whole-genome sequences of ASFV.

## ASFV whole-genome sequencing—History and state of play

**Fig 1:**
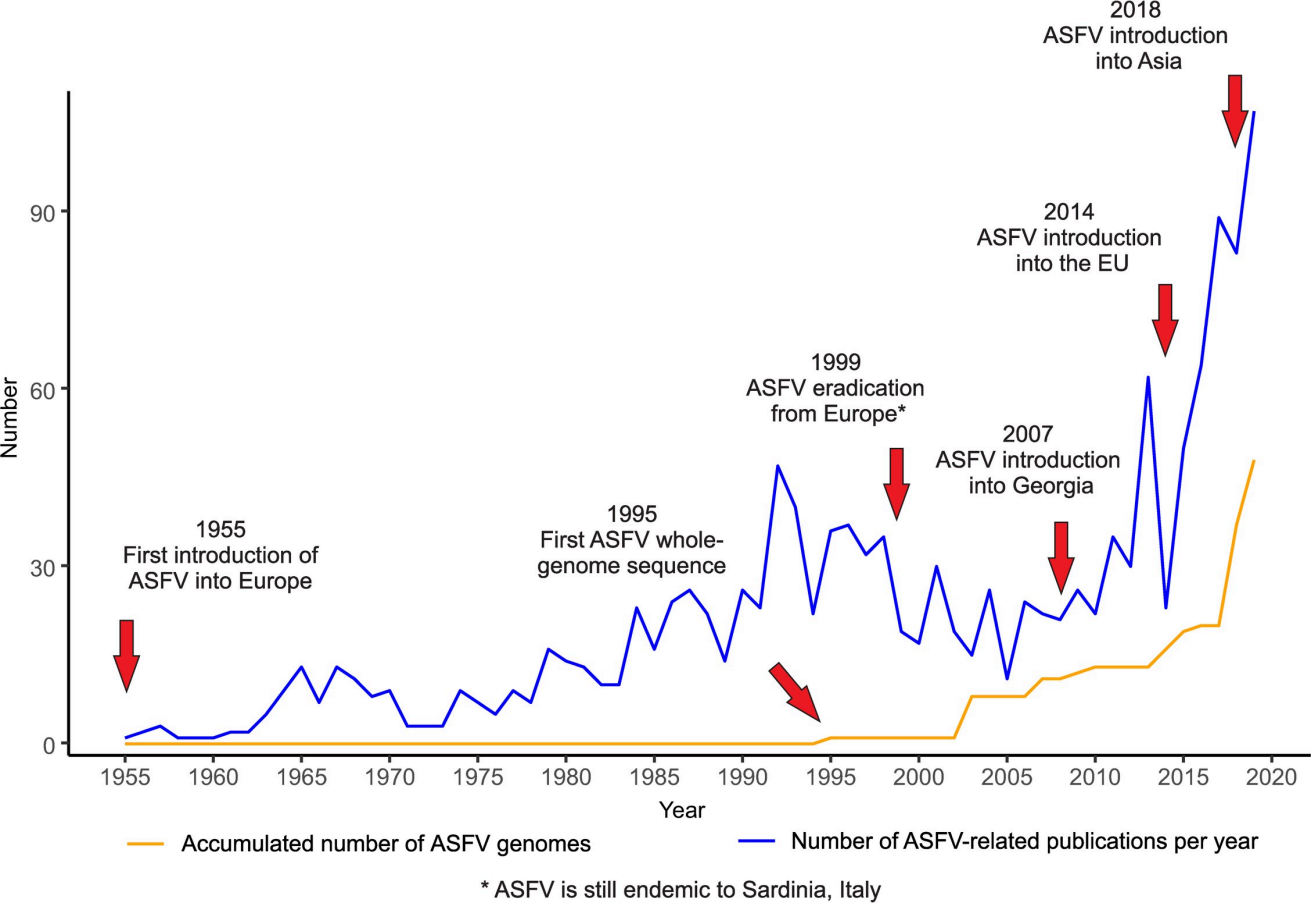
ASFV-related publications and whole-genome sequences over time. The number of publications related to ASFV per year (blue line) was extracted from NCBI-Pubmed on December 20, 2019, and the accumulated number of ASFV whole-genome sequences (orange line) was extracted from INSDC databases on December 20, 2019. Important events are highlighted (red arrows). ASFV, African swine fever virus; EU, European Union; INSDC, International Nucleotide Sequence Database Collaboration; NCBI, National Center for Biotechnology Information.

In 1995, the first whole-genome sequence of cell-culture–adapted ASFV strain BA71V was published, providing the first complete genome information on ASFV and therefore a basis for in-depth characterisation [1,6]. However, because whole-genome sequencing using the existing techniques was still extremely laborious, and research interest—due to the eradication of ASF from Europe—decreased, only few additional whole genomes were published in the following years (Fig 1).

**Fig 2:**
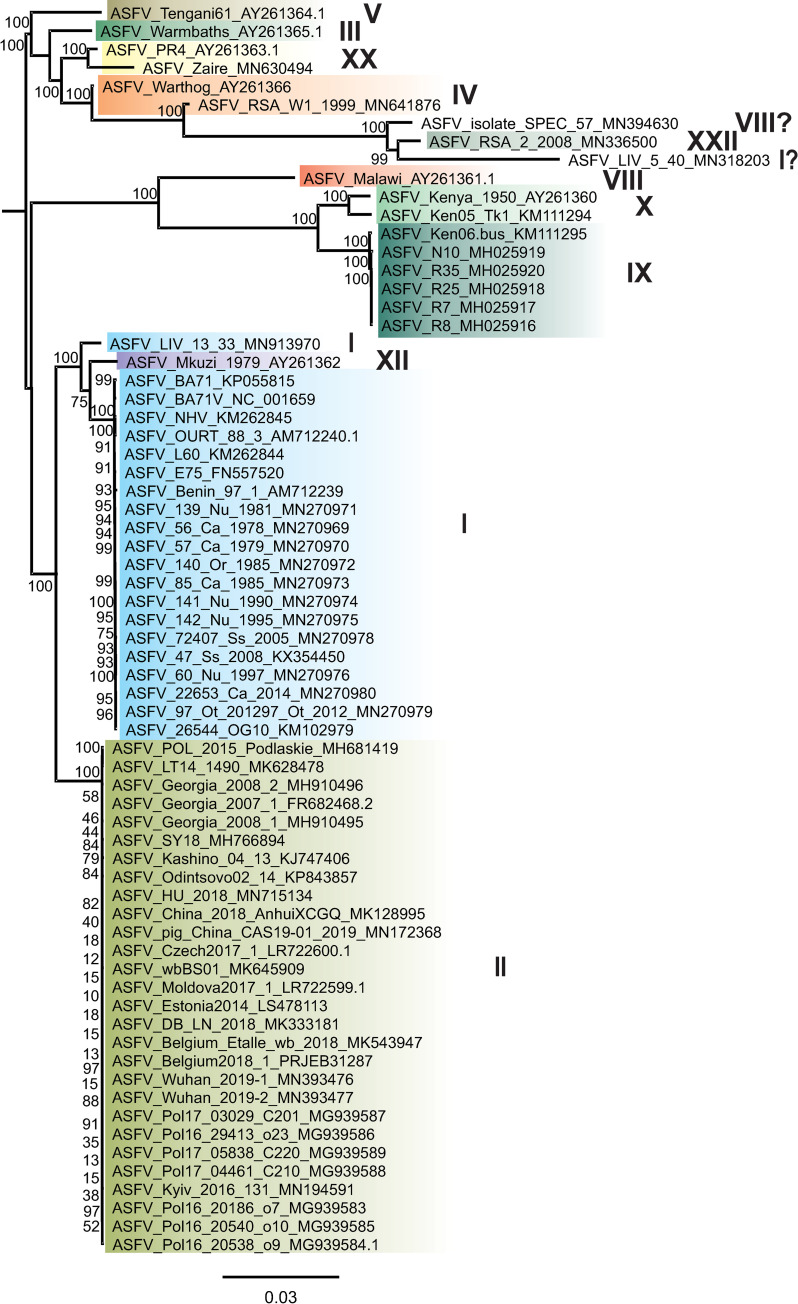
Phylogenetic reconstruction using all publicly available ASFV whole-genome sequences. The ML tree was constructed using IQ-TREE version 1.6.5 based on MAFFT version 7.388 aligned ASFV core-regions (approximately 137,000 bp from the 5′-A224 to the 3′-DP238L gene) downloaded from INSDC (status 13.05.2020). Standard model selection was used, resulting in the best-fit model GTR+F+R4 (general time reversible model with unequal rates and unequal base frequency + empirical base frequencies + FreeRate model with 4 categories). Statistical support of 10,000 ultrafast bootstraps using the ultrafast bootstrap approximation (UFBoot) (percentage values) is indicated at the nodes. Taxon names include, where available, ASFV designation and INSDC accession number and P72 GT. The scale bar represents number of substitutions per site. The update of the ASFV Georgia 2007/1 sequence from FR682468.1 to FR682468.2 is currently in progress, and FR682468.2 will be available from INSDC soon. The accession LR743116.1, wrongly assigned to the updated ASFV Georgia 2007/1 sequence, was removed from the databases. ASFV, African swine fever virus; GT, genotype; INSDC, International Nucleotide Sequence Database Collaboration; ML, maximum likelihood.

When ASF re-emerged in 2007, research interest into ASFV increased drastically (Fig 1) [2]. Together with technical advances in sequencing, e.g., the use of second-generation high-throughput sequencing platforms [7], a few additional whole-genome sequences were published and used as a basis for genetic characterisation, virus comparison, and vaccine development [7]. However, only after the introduction of ASF into the EU in 2014 did ASF become a research priority. Since then, numerous ASFV sequences of the respective strains have been published using the latest sequencing methods with the goal to identify genetic markers and trace routes of introduction through molecular epidemiology (Figs 1 and 2) [8]. With the introduction of ASF into Asia, home of the most dense population of domestic pigs in the world, the world is now facing the worst pandemic of an animal disease seen to date, and new ASFV whole-genome sequences from Europe and Asia are being published with increasing frequency (Figs 1 and 2) [9]. Nevertheless, we have to ask ourselves whether resources are well invested since the knowledge gained from recently published ASF genome sequences does not meet the expectations.

## What do we expect from sequencing ASFV?

Expectations are high when it comes to whole-genome sequences of ASFV. As observed for other pathogens, whole-genome sequence information is expected to help unravelling disease pattern with molecular epidemiology, assist in tracing outbreaks, foster the understanding of virulence, and create the basis for the design of tailored diagnostic tools and vaccine development.

## Do we meet those expectations?

As of today (May 13, 2020), 70 ASFV whole-genome sequences are publicly available (Fig 2). While at the first glance this looks like plentiful material to work with, a deeper investigation shows problems concerning quantity and quality.

### Quantity

Through partial sequencing of the ASFV B646L gene, coding for the major structural protein P72, 24 genotypes (GTs) have been identified so far [10]. However, the 70 available ASFV whole-genome sequences cover only 13 of these GTs with a bias toward the 2 pandemic genotypes I (GTI) (21 sequences) and II (GTII) (29 sequences) (Fig 2). Thus, strains circulating between the natural hosts in the sylvatic cycle are underrepresented (Fig 2).

### Quality

Bioinformatic analyses of ASFV sequences are aggravated by genome complexity. Artefacts are, e.g., caused by using non-suited bioinformatics workflows or sequencing platforms leading to low coverage and misassemblies in the extensive homopolymer and repeat regions [8]. Given these problems in combination with the lack of data regarding quality parameters [11], methodology, and outdated annotations, many published sequences are not suitable for detailed analyses.

## Do we need better methods?

Today, high-throughput sequencing platforms of the second generation that produce short reads (50–500 bp) with high accuracy provide good and reliable results. However, the enormous amount of host sequences in the background of most samples leads to a very low virus-to-host-sequence ratio. For ASFV, shotgun sequencing of untreated organ samples usually provides around 0.05%–0.1% viral reads, while datasets from cell-culture supernatant can contain 1%–5% [8]. To assemble a reliable complete genome sequence (mean coverage of around 50), a minimum of around 60,000 single reads (150 bp) is required. Therefore, shotgun sequencing of organ samples but also tissue culture samples require high sequencing capacity of many million reads per sample leading to huge datasets that need to be handled and stored.

To overcome this obstacle, methods for target specific enrichment and host depletion have been implemented and successfully used for ASFV sequencing [8,12]. Employing these techniques has led to a significantly higher virus-to-host ratio with 25%–60% viral reads per dataset. However, even with these techniques, sequencing in homopolymer and repeat regions is still challenging. Therefore, third-generation sequencing platforms such as MinION (Oxford Nanopore Technologies) and PacBio (Pacific Biosciences of California) sequencing producing single-molecule ultra-long reads have been employed [8,13]. Although the accuracy, especially for the Nanopore reads, is usually low, they can provide a backbone that—in combination with the short-read data—allows for the assembly of very high-quality ASFV whole-genome sequences [8]. However, identifying and verifying single-nucleotide differences and variants is still challenging, and—especially in the long homopolymer stretches of up to 16 G and 17 C nucleotides and extensive inverted terminal repeat regions at the genome ends—the existing methods reach their limit.

## Are we on the wrong path?

Huge financial and technical resources have been dedicated to ASFV whole-genome sequencing to provide a basis for molecular epidemiology for the current pandemic. However, although 29 whole-genome sequences from the corresponding GTII strains from 10 affected countries have been published in the last 10 years, only 2 regions showing significant differences were identified [14], and none of those have proven useful for larger-scale molecular epidemiology or source tracking so far. Instead, the sequences show more than 99.9% nucleotide sequence identity and differ in only very few single nucleotides distributed over the entire genome without a clear pattern or related change in phenotype which cannot be easily distinguished from sequencing or bioinformatics artefacts [8]. Therefore, most of the ASFV GTII whole-genome sequences published since 2010 do not provide any additional information useful to understand virus evolution or combat the disease, and the financial and technical resources have been more or less wasted.

## Is it still worth trying?

Despite the high identity of circulating ASFV GTII strains, rare genetic variants have been observed. These variants include strains with single-nucleotide changes affecting their phenotype [15] as well as viruses showing large genome reorganisations and deletions [16]. Since these variants offer a great opportunity to learn about virus evolution and gene functions, it is imperative to identify and analyse them. Therefore, samples for sequencing should be chosen carefully and prioritised for samples from outbreaks where unusual patterns have been observed, for example, a lower virulence.

Furthermore, to use the available resources most efficiently, the current system of expensive and laborious in-depth sequencing of ASFV strains needs to be changed towards a more targeted approach. Here, novel methods—e.g., the combination of target enrichment by hybridisation capture and multiplex sequencing using Nanopore sequencing or small-scale Illumina platforms (iSeq 100)—could provide a quick and affordable alternative for screening multiple viral genomes for variations followed by in-depth characterisation of selected candidates.

In addition, more virus strains from Africa should be sequenced to elucidate ASFV evolution and mechanisms of genetic adaptation as well as the emergence of novel GTs and prepare for the future spread of other ASFV GTs that, due to limited cross-protection in vaccinated animals (with a future vaccine against ASFV GTII), might require different intervention strategies.

Therefore, cooperation with researchers based in Africa is essential to join in solving this global problem.

But not only field samples should be considered. ASFV strains that were sequenced in the past need to be checked and validated using the most up-to-date sequencing methods to remove sequencing or bioinformatics artefacts making them useful for comparative in-depth analyses. Furthermore, strains that were passaged many times should be analysed to validate their genome integrity prior to the use in experimental studies, and cell-line adapted strains as well as genetically modified variants should also be checked very carefully for off-target effects.

In conclusion, whole-genome sequencing is an essential tool in understanding this extraordinary pathogen and the basis for vaccine development. However, efforts must be made to optimise and harmonise protocols for sample selection, sequencing, and bioinformatics workflows as well as documentation and sharing of data (including raw reads) to use the financial and technical resources most efficiently and generate valuable data—data that are desperately needed to stop one of the most devastating animal pathogens of our time.

## References

[1] Arias M, Sánchez-Vizcaíno JM. African Swine Fever Eradication: The Spanish Model In: Morilla A, Yoon KJ, Zimmerman JJ, editors. Trends in Emerging Viral Infections of Swine, 1. USA: Iowa State Press; 2002 p. 133–9.

[2] Sanchez-Vizcaino JM, Mur L, Martinez-Lopez B. African swine fever (ASF): five years around Europe. Veterinary microbiology. 2013;165(1–2):45–50. 10.1016/j.vetmic.2012.11.030 23265248

[3] FAO. ASF situation in Asia update 2019. [2020 May 17]. Available from: http://www.fao.org/ag/againfo/programmes/en/empres/ASF/Situation_update.html.

[4] Smith D, Cooper T, Pereira A, Jong J. Counting the cost: The potential impact of African Swine Fever on smallholders in Timor-Leste. One Health. 2019;8:100109 10.1016/j.onehlt.2019.100109 31687470 PMC6819879

[5] Pitts N, Whitnall T. Impact of African swine fever on global markets: Australian Government, Department of Agriculture (ABARES); 2019 [2020 May 17]. Available from: https://www.agriculture.gov.au/abares/research-topics/agricultural-commodities/sep-2019/african-swine-fever.

[6] Yanez RJ, Rodriguez JM, Nogal ML, Yuste L, Enriquez C, Rodriguez JF, et al Analysis of the complete nucleotide sequence of African swine fever virus. Virology. 1995;208(1):249–78. 10.1006/viro.1995.1149 11831707

[7] Chapman DA, Darby AC, Da Silva M, Upton C, Radford AD, Dixon LK. Genomic analysis of highly virulent Georgia 2007/1 isolate of African swine fever virus. Emerg Infect Dis. 2011;17(4):599–605. 10.3201/eid1704.101283 21470447 PMC3379899

[8] Forth JH, Forth LF, King J, Groza O, Hübner A, Olesen AS, et al A Deep-Sequencing Workflow for the Fast and Efficient Generation of High-Quality African Swine Fever Virus Whole-Genome Sequences. Viruses. 2019;11(846):1–18. 10.3390/v11090846 31514438 PMC6783980

[9] Zhou X, Li N, Luo Y, Liu Y, Miao F, Chen T, et al Emergence of African Swine Fever in China, 2018. Transbound Emerg Dis. 2018;65(6):1482–4. 10.1111/tbed.12989 30102848

[10] Quembo CJ, Jori F, Vosloo W, Heath L. Genetic characterization of African swine fever virus isolates from soft ticks at the wildlife/domestic interface in Mozambique and identification of a novel genotype. Transbound Emerg Dis. 2018;65(2):420–31. 10.1111/tbed.12700 28921895 PMC5873395

[11] Ladner JT, Beitzel B, Chain PSG, Davenport MG, Donaldson EF, Frieman M, et al Standards for sequencing viral genomes in the era of high-throughput sequencing. mBio 2014;5(3): e01360–14. 10.1128/mBio.01360-14 24939889 PMC4068259

[12] Masembe C, Sreenu VB, Da Silva Filipe A, Wilkie GS, Ogweng P, Mayega FJ, et al Genome Sequences of Five African Swine Fever Virus Genotype IX Isolates from Domestic Pigs in Uganda. Microbiol Resour Announc. 2018;7(13):e01018–18. 10.1128/MRA.01018-18 30533685 PMC6256554

[13] Granberg F, Torresi C, Oggiano A, Malmberg M, Iscaro C, De Mia GM, et al Complete Genome Sequence of an African Swine Fever Virus Isolate from Sardinia, Italy. Genome Announc. 2016;4(6):e01220–16. 10.1128/genomeA.01220-16 27856577 PMC5114369

[14] Gallardo C, Fernandez-Pinero J, Pelayo V, Gazaev I, Markowska-Daniel I, Pridotkas G, et al Genetic variation among African swine fever genotype II viruses, eastern and central Europe. Emerg Infect Dis. 2014;20(9):1544–7. 10.3201/eid2009.140554 25148518 PMC4178389

[15] Gallardo C, Soler A, Rodze I, Nieto R, Cano-Gomez C, Fernandez-Pinero J, et al Attenuated and non-haemadsorbing (non-HAD) genotype II African swine fever virus (ASFV) isolated in Europe, Latvia 2017. Transbound Emerg Dis. 2019;66(3):1399–404. 10.1111/tbed.13132 30667598

[16] Zani L, Forth JH, Forth L, Nurmoja I, Leidenberger S, Henke J, et al Deletion at the 5’-end of Estonian ASFV strains associated with an attenuated phenotype. Scientific reports. 2018;8(6510):1–11. 10.1038/s41598-018-24740-1 29695831 PMC5916933

